# A Fast Soft Continuum Catheter Robot Manufacturing Strategy Based on Heterogeneous Modular Magnetic Units

**DOI:** 10.3390/mi14050911

**Published:** 2023-04-23

**Authors:** Tieshan Zhang, Gen Li, Xiong Yang, Hao Ren, Dong Guo, Hong Wang, Ki Chan, Zhou Ye, Tianshuo Zhao, Chengfei Zhang, Wanfeng Shang, Yajing Shen

**Affiliations:** 1 The Robot and Automation Center and the Department of Biomedical Engineering, City University of Hong Kong, Kowloon, Hong Kong 999077, China; 2 Shenzhen Research Institute, City University of Hong Kong, Shenzhen 518057, China; 3 The Department of Electronic and Computer Engineering, Hong Kong University of Science and Technology, Kowloon, Hong Kong 999077, China; 4 Research Center on Smart Manufacturing, Hong Kong University of Science and Technology, Kowloon, Hong Kong 999077, China; 5 Prince Philip Dental Hospital, Faculty of Dentistry, University of Hong Kong, Hong Kong 999077, China; 6 Applied Oral Sciences and Community Dental Care, Faculty of Dentistry, University of Hong Kong, Hong Kong 999077, China; 7 The Department of Electrical and Electronic Engineering, University of Hong Kong, Hong Kong 999077, China; 8 Guangdong Provincial Key Laboratory of Robotics and Intelligent System, Shenzhen Institute of Advanced Technology, Chinese Academy of Sciences, Shenzhen 518055, China

**Keywords:** modular fabrication, magnetic continuum robots, modular robots, biomedical applications

## Abstract

Developing small-scale continuum catheter robots with inherent soft bodies and high adaptability to different environments holds great promise for biomedical engineering applications. However, current reports indicate that these robots meet challenges when it comes to quick and flexible fabrication with simpler processing components. Herein, we report a millimeter-scale magnetic-polymer-based modular continuum catheter robot (MMCCR) that is capable of performing multifarious bending through a fast and general modular fabrication strategy. By preprogramming the magnetization directions of two types of simple magnetic units, the assembled MMCCR with three discrete magnetic sections could be transformed from a single curvature pose with a large tender angle to a multicurvature S shape in the applied magnetic field. Through static and dynamic deformation analyses for MMCCRs, high adaptability to varied confined spaces can be predicted. By employing a bronchial tree phantom, the proposed MMCCRs demonstrated their capability to adaptively access different channels, even those with challenging geometries that require large bending angles and unique S-shaped contours. The proposed MMCCRs and the fabrication strategy shine new light on the design and development of magnetic continuum robots with versatile deformation styles, which would further enrich broad potential applications in biomedical engineering.

## 1. Introduction

Small-scale soft magnetic robots, with the advantages of good controllability, rapid response, and safety, were widely investigated [1,2,3,4,5,6], especially regarding their great prospects in biomedical applications [7,8], such as targeted drug delivery and minimally invasive surgery. Specifically, magnetic soft robots can be divided into untethered and tethered types. The former has a wireless actuation form that allows it to enter biological bodies and implement biomedical operations in vivo under the control of an external magnetic field [9,10,11,12,13,14,15]. The latter is often combined with conventional continuum robots in a tethered manner to perform interventional surgery [16,17,18]. Unlike wireless magnetic robots that need to consider their own biosafety, tethered robots can be directly retrieved without residues. In a recent proof of concept [19] for solving practical biomedical issues [20], tethered magnetic continuum robots showed great potential in accessing hard-to-reach lesions to perform minimally invasive surgeries. Such progress could benefit versatile biomedical applications [21,22] (including targeted drug delivery, sampling, and diseased tissue ablation) in next-generation precision medicine.

Many actuation principles were proposed for continuum robots [23], such as tendon- [24,25,26,27], fluid- [28,29,30], and smart-material-driven [31,32]. The strengths of magnetic continuum robots include easily achieving a small-scale contour [19,33], being actuated with uncomplicated external operation systems [34], and capable of controlling with high precision [20,35]. None of the other driving mechanisms can have all these advantages. Therefore, magnetic-responsive continuum robots provide great opportunities for examination and treatment in narrow spaces in the human body, e.g., the lungs and veins or arteries. In order to respond to a magnetic field, magnetic continuum robots usually contain hard micromagnets [36] or magnetic-microparticle-embedded soft polymers [19]. However, magnetic continuum robots with micromagnets encounter difficulty in enabling the entire body to comply despite a relatively more obvious magnetic response. A polymer-based magnetic continuum robot with an inherent soft body has superior flexibility and miniaturization [19,20,36,37]. However, soft continuum robots with single magnetic response section usually show the ability to actively steer in their distal tip with little pose control capacity for the long rear body, meeting challenges in accessing more complex channel environments. To construct a magnetic continuum robot with more controllable bending modes, multiple micromagnets are discretely integrated into the forebody [16,17,38], reducing compliance. To establish a fully soft body, a magnetic continuum robot with pose control can be produced through specifically integrated [39] and heat-assisted [40] magnetization. However, the above methods involve complex auxiliary processing equipment, e.g., specified molds and lasers, hindering rapid mass fabrication. Therefore, considering the diverse scenarios that may occur in actual operation tasks, a simple and general fabrication strategy is necessary for quickly constructing soft magnetic continuum catheter robots with the expected bending features.

**Figure 1:**
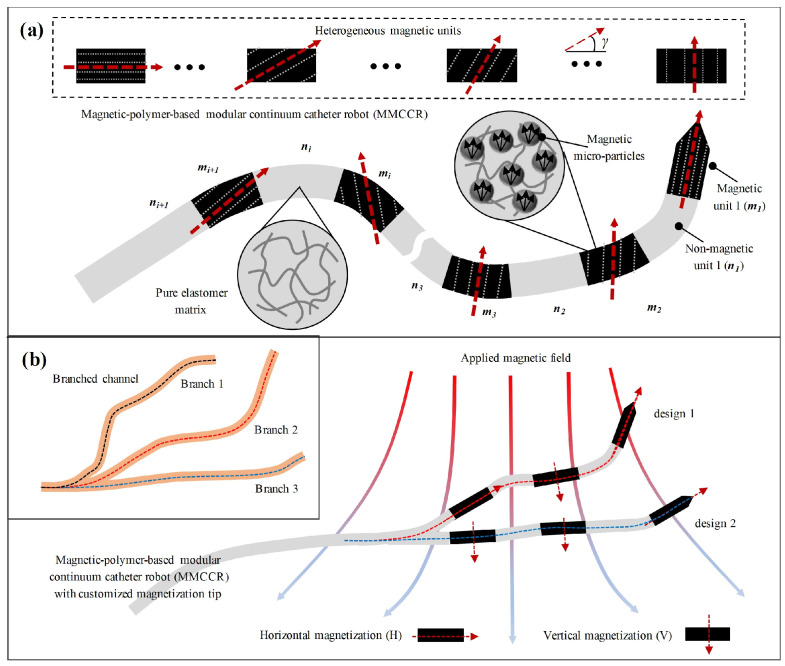
Programmable magnetic-polymer-based modular continuum catheter robot (MMCCR) (**a**) consisting of multiple heterogeneous modular units. (**b**) MMCCRs with three customized magnetization units for accessing different branched channels.

In this work, we present a magnetic-polymer-based modular continuum catheter robot (MMCCR) that could realize rich bending modes after quick modular assembly (Figure 1a). The MMCCR contains multiple heterogeneous modular units with/without a magnetic response. The magnetic units (composed of soft polymer and iron microparticles) were designed with different fixed magnetization directions. Unlike previous studies that fabricated magnetic continuum robots with hard magnets, the embedded magnetic sections in our model are soft in terms of mechanical properties, and their magnetization direction could be preprogrammed, which allows for designing the MMCCR with multifarious bending modes. To verify such a concept, we first constructed multiple MMCCRs with three sections on the basis of two types of magnetized units. We show and analyze the static and dynamic deformation performance of the MMCCRs. Lastly, we demonstrate a branched channel access to illustrate the adaptive design of the MMCCs. This study enriches the research on magnetic continuum robots and could benefit their usage in extensive medical operations.

## 2. Materials and Methods

### 2.1. MMCCR Design

To achieve pose control for the whole body, a soft magnetic continuum catheter robot is given discrete magnetization directions. Except for integrated molding technology and laser-assisted magnetization, a modular design is another prime approach to achieve a customized magnetic response for a soft continuum catheter robot. As shown in Figure 1a, an MMCCR consists of multiple heterogeneous magnetic units connected with nonmagnetic units, where nonmagnetic units (*n_i_*) are composed of a pure elastomer matrix, and magnetic units (*m_i_*) are with a magnetized microparticle composition embedded in the matrix. The MMCCR showed a fully soft body in the elastomer matrix for smoothly interacting with the environment. The number of discrete magnetic units and the corresponding magnetization directions (angle *γ* in the range of 0–90 degrees) could be changed to achieve an adaptive curvature through customized design according to the application scenarios.

To demonstrate the concept of the MMCCRs, robots with three magnetic units were designed on the basis of two magnetization directions, i.e., horizontal (H) and vertical (V), as shown in Figure 1b. By assembling the magnetic units in different styles, the MMCCRs could be actuated with the magnetic field to adapt different channels. Figure 1b shows that Robot Designs 1 and 2 correspond to the H–V–H and H–V–V magnetization arrangements, respectively, from the tip to the rear body. With an applied magnetic field, Robots 1 and 2 could be deformed into a specialized pose status for Branches 2 and 3, respectively. To reduce the potential contact resistance and thereby enhance the advancing capability, we designed a conical magnetic tip for the robot (see Figure S1 and Table S1 for detailed dimensional design). With both the geometrical design and magnetization direction preprogramming, the proposed MMCCR could easily access varied channel environments under remote magnetic actuation.

### 2.2. MMCCR Fabrication

**Figure 2:**
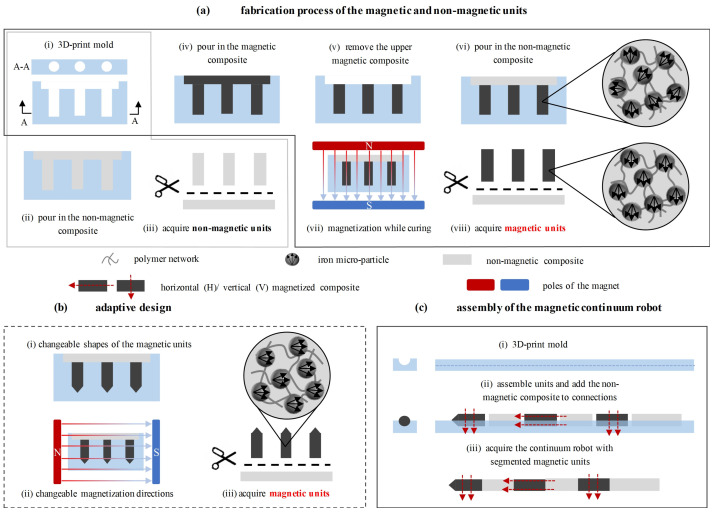
Fabrication process of the MMCCR. (**a**) General fabrication process of magnetic and nonmagnetic units. (**b**) Adaptive design and corresponding fabrication of magnetic units. (**c**) MMCCR assembly procedure.

To achieve the rapid mass fabrication of such an MMCCR, a molding technology was applied as the main method, and silicone rubber with/without iron microparticles was utilized to construct the soft body. First, as shown in Figure 2a–i, molds with multiple long holes were printed with a micro 3D printing system (Form 3+, Formlabs Inc., Somerville, MA 02143–02145, USA) with printing precision of 50 μm. Then, Precursors A and B of the silicone rubber, i.e., Dragonskin #20 (Smooth-on Inc., Macungie, PA 18062, USA) were mixed evenly at a weight ratio of 1:1 and vacuumed to reduce air bubbles within. Third, the mixture was poured into the mold and cured under 70 °C for 30 min (Figure 2a(ii)). The nonmagnetic units of the MMCCR were obtained by cutting the soft pillars out (Figure 2a(iii)). The magnetic units could be similarly fabricated. By adding iron microparticles (Guangzhou Metallurgy Co., Ltd., Guangzhou, China) into the precursor mixture that had an A:B:iron weight ratio of 1:1:1.5, the magnetic composite was obtained. Then, the magnetic mixture was poured into the mold (Figure 2a(iv)), and the upper layer was removed after 5 min of vacuuming (Figure 2a(v)). Then, a layer of a nonmagnetic composite was used as a cover (Figure 2a(vi)), and the mixture was cured under 70 °C for 30 min while a uniform magnetic field was applied (Figure 2a(vii)). During curing, the embedded iron microparticles were rearranged to align them with the applied magnetic field (as shown in the magnified subfigure). Lastly, the magnetic units with horizontal magnetization direction could be cut out (Figure 2a(viii)). To achieve variable poses and adaptation to different environments, the discrete section could also be molded into varied contours and magnetized into diverse directions. Here, we demonstrate an adaptive designs for a magnetic unit with a conical tip (Figure 2b(i)) and vertical magnetization direction (Figure 2b(ii/iii)). Once all the magnetic/nonmagnetic units had been obtained, the magnetic continuum robot could be assembled. As shown in Figure 2c, multiple units with/without magnetic response could be connected after curing with the same nonmagnetic composite. Lastly, MMCCRs were obtained with a diameter of 3 mm for the main body and a conical tip of around 1 mm.

With the obtained batch of magnetic/nonmagnetic modular units through molding, different MMCCRs could be quickly fabricated.

### 2.3. Characterization of the Applied Magnetic Field

**Figure 3:**
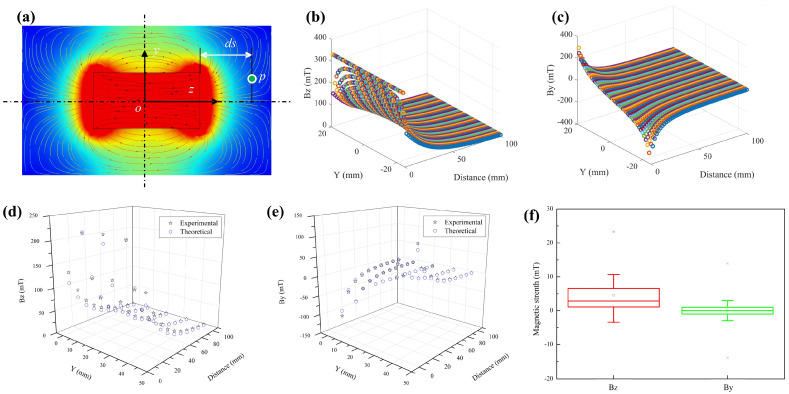
Characterization of the magnetic field source. (**a**) Simulation results of the magnetic field distribution of a magnet. Theoretical results of gradient magnetic field strength along the (**b**) Bz and (**c**) By axes. Comparison of the (**d**) theoretical and (**e**) experimental results of the gradient magnetic field. (**f**) Box map of the magnetic strength (Bz/By) difference between the theoretical and experimental results.

To control the proposed MSCR, a gradient magnetic field was first established and characterized. Four Nd–Fe–B magnets (N52, Shanghai Yanti Metal Materials Co., Ltd., Shanghai, China) with dimensions of 45 × 45 × 20 mm were serially connected as the magnetic field source. To characterize the generated magnetic field, the planar distribution (*y–o–z*) of a magnet (45 × 45 × 80 mm) was first simulated in COMSOL Multiphysics software (v. 5.4), as shown in Figure 3a. According to the thermal map and virtual magnetic induction lines (i.e., the curved arrows), the magnetic strength was symmetrical with respect to the *x–o–z* plane and decreased with the increase in distance; the arrow density showed symmetrical distribution and became more dispersed. Setting a point P with a distance of *ds* from the right surface of the magnet, the theoretical magnetic field strength of that point could be computed on the basis of a permanent magnet model [41]. The calculated distribution in the *y–o–z* plane along the Bz and By axes against the y coordinate and distance *ds* are found in Figure 3b,c, respectively. Magnetic strength along the Bz axis was symmetrical with respect to the *x–o–z* plane, while By strength was symmetrical on the basis of the *z* axis. The magnitude of the two gradually decreased as the distance increased. The trend of the calculated magnetic field distribution was the same as that in the simulated results (Figure 3a).

To confirm the effectiveness of the simulation and computation, the gradient magnetic strength of the source was also experimentally measured. With a teslameter, a 3-DOF platform, and two lifting platforms, magnetic strength along the Bz and By axes was measured with the deployed z/y-directional probe; the experimental setup is shown in Figure S2a,b. The experimental planar distribution (*y–o–z*) and the corresponding theoretical value are presented in Figure 3d,e. The theoretical and experimental trends were the same with a slight difference shown in Figure 3f, where the median and 50% fluctuation of the error were smaller than 5 and 10 mT, respectively. We could generally verify the established theoretical model’s accuracy.

### 2.4. Test Model Preparation

To characterize the bending performance of the MMCCRs, the obtained MMCCR was fixed to the forehead of a 3-DOF platform while maintaining it inside the *y–o–z* plane of the magnetic source; Figure S2c shows the experimental setup. The conical tip of the robot was kept inside the *x–o–z* plane of the magnet, where it hanged freely at a distance of 85 mm from the magnet surface. The displacement of the MMCCR was controlled by manually manipulating the *x*-directional platform. Then, the mass center *P_i_* (*i* = 1, 2, 3) and bending angle *α_i_* (*i* = 1, 2, 3) of the three magnetic units *m_i_* (*i* = 1, 2, 3) could be obtained (Figure S2d). To perform the quantitative analysis of the bending performance of the robot, a series of image processing procedures were performed to extract the positional and pose information of the magnetic units, as shown in Figure S3.

To perform an adaptive navigation demonstration, a bronchial tree phantom was printed with transparent resin (Figure S5). By placing the branched channels at different orientations with respect to the outlet of the left fourth bronchus, the adaptive access capacity of the MMCCRs is proved.

## 3. Results

### 3.1. Static Bending Analysis of MMCCRs

To better adapt to different environments, the proposed MMCCR could be preprogrammed into varied magnetization arrangements. According to the aforementioned fabrication procedure, 8 different deployment schemes S*_i_* (*i* = 1, 2, 3, 4, 5, 6, 7, 8) for the three-sectional MMCCR were produced on the basis of the horizontal (H)/vertical (V) magnetized magnetic unit shown in Table S2.

**Figure 4:**
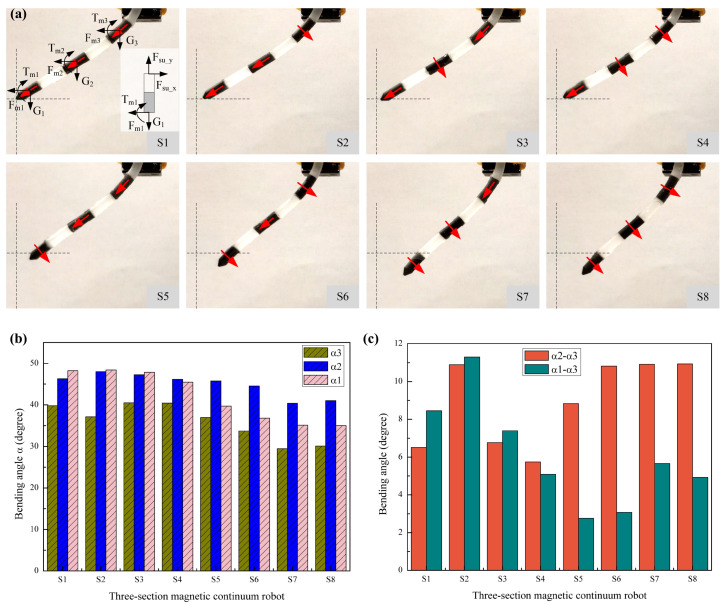
Bending status analysis of the MMCCRs. (**a**) Final pose status of all 8 types of MMCCRs under magnetic actuation. (**b**) Statistical comparison of the bending angles of the 8 schemes. (**c**) Bending-angle difference of the two lower sections (*α*_1_, *α*_2_) with respect to the base section (*α*_3_).

The static bending status with a displacement of 25 mm relative to the origin of the 8 types of MMCCRs is shown in Figure 4a. The entire bending deformation of the downward free body shrank with the scheme shift, and the contour of the MMCCRs became more S-shaped. The extracted pose information of the three magnetic units at this moment is shown in Figure 4b. According to the bar chart, it can be concluded that all three angles experience a rough decrease as the scheme changes from S1 to S8. The bending angle of the first three schemes from the fixing section to the tip, i.e., α_3_ to α_1_, showed an increasing trend that suggested that the bending performance of these MMCCRs could be simplified into a single curvature deformation. However, angles α_1_ and α_2_ experienced an exchange in the numerical sequence after Scheme S4, i.e., α_2_ became the largest angle, which suggests that an S-shaped multicurvature deformation occurred in the MMCCR actuation. The bending-angle difference of the two lower sections (α_1_, α_2_) with respect to the base section (α_3_) was further analyzed, as shown in Figure 4c. The difference suddenly increased to 10.89 and 11.29 degrees when the scheme changed from S1 to S2, i.e., base magnetic section m3 was replaced with a vertical magnetization unit. The difference shrank in Schemes S3 and S4. In the last four schemes, the angle (α2–α3) was obviously almost twice larger than the angle difference between the tip and base section. Scheme S6 had the most apparent difference, namely, 10.81 degrees for *α*_2_–*α*_3_ and 3.07 degrees for *α*_1_–*α*_3_, which suggests the greatest curvature in the middle body of the MMCCR.

Considering the generated gradient magnetic field, the magnetic units were subject to both magnetic force and magnetic torque, as shown in Figure 4a. The magnetic unit was set with magnetic moment *m* in its center and positioned inside magnetic field *B*. Magnetic force *F_mi_* and torque *T_mi_* can be expressed as follows:

(1) $$F_{mi}=\left(m\cdot \nabla \right)B$$

(2) $$T_{mi}=m\times B$$

The subjected magnetic force *F_mi_* attracted each magnetic unit closer to the magnetic source, while magnetic torque *T_mi_* induced a pose shift, steering the inherent magnetization direction to align with the virtual magnetic induction line. If the downward free body of the MMCCR was segmented into three sections (Figure 4a), and the section’s mass point coinciding with the magnetic unit was simplified, the mechanical equilibrium of each section could be presented as follows:

(3) $$\sum Fx=F_{mi}-F_{su\_x}=0$$

(4) $$\sum Fy=G_{i}-F_{su\_y}=0$$

(5) $$\sum T=-\left(T_{mi}+F_{mi}l_{effect}\cos\alpha_{i}\right)+T_{su}+G_{i}l_{effect}\sin\alpha_{i}=0$$

where *G_i_, F_su_x_*, *F_su_y_*, and *T_su_* denote the simplified gravity, the support reaction force along the *x*/*y* axis, and the support reaction torque, respectively; $l_{effect}=l_{n1}+l_{m1}/2$ represents the effective force arm of each section.

To analyze the deformation, the bottommost section was taken as an example and viewed as a cantilever beam with length $l_{effect}$. Gravity *G*_1_ and magnetic force/torque *F_m_*_1_/*T_m_*_1_ were simplified to the tip of the beam, and the latter two could be further simplified into point force $F_{effect1}=F_{m1}+F_{T1}$, where $F_{T1}$ denotes the equivalent force of the subjected magnetic torque. Then, the generated shear force at arc length *l* is expressed as follows:

(6) $$Fs=F_{effect1}l\cos\alpha_{1}(l)-G_{1}l\sin\alpha_{1}(l)$$

where $\alpha_{1}(l)$ denotes the deflection angle at arc length *l* induced by force. The entire deflection angle of this section is represented as follows:

(7) $$EI\alpha_{1}=G_{1}l_{effect}\sin\alpha_{1}-F_{effect1}l_{effect}\cos\alpha_{1}-G_{1}l_{effect}^{2}+F_{effect1}l_{effect}$$

According to Equation (7), the deflection angle could be generally viewed as an inverse proportion function of the elastic modulus, i.e., $\alpha_{i}\propto \left(1/E\right)$. The three-section MMCCR is the combination of multiple cantilever beams, so the entire deflection at the tip is expressed as follows:

(8) $$\alpha =\sum_{i=1}^{3}\alpha_{i}$$

To better understand the above deformation, the mechanical analysis of the three typical schemes (i.e., S2, S6, and S8) is also shown in Figure S4. The key difference is the direction of the magnetic torque to which each section was subjected, and the simplified point force can be expressed as $F_{effecti}=F_{mi}\pm F_{Ti}$, where the sign depends on the direction of the magnetic torque in each section. The + indicates a torque that was in the same direction as that of the bending, while a—denotes a torque in the opposite bending direction.

On the basis of the static deformation of the MMCCRs, Schemes S1 to S3 were more suitable for large tender angle steering tasks, while the five other schemes were better for multicurvature environment navigation.

### 3.2. Dynamic Deformation of MMCCRs

**Figure 5:**
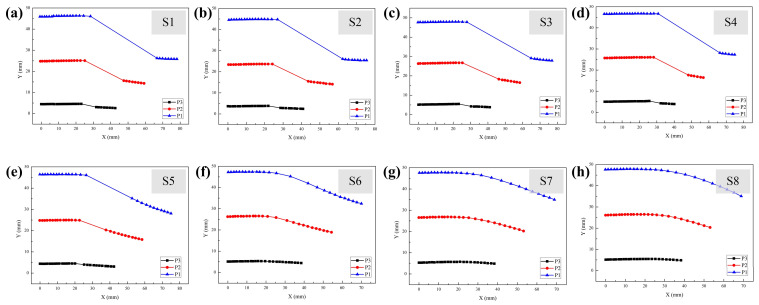
Extracted mass center position information of the 3 magnetic units for schemes (**a**) S1, (**b**) S2, (**c**) S3, (**d**) S4, (**e**) S5, (**f**) S6, (**g**) S7, and (**h**) S8 of MMCCRs.

**Figure 6:**
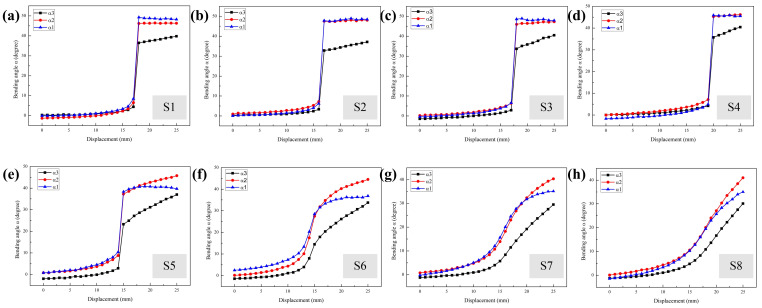
Extracted pose information of 3 magnetic units for schemes (**a**) S1, (**b**) S2, (**c**) S3, (**d**) S4, (**e**) S5, (**f**) S6, (**g**) S7, and (**h**) S8 of MMCCRs.

To evaluate the dynamic deformation of the MMCCRs under magnetic actuation, the robots were manually moved from an 85 mm distance to a 25 mm displacement relative to the origin. The bending status of the MMCCR was recorded at every 1 mm step. Information regarding the mass center position and the pose of the three magnetic units during the moving process is shown in Figure 5 and Figure 6, respectively.

Figure 5 shows that all 8 MMCCR schemes experienced a decrease in the y coordinate as the x coordinate increased, which suggests that the entire MMCCR body was bending. Schemes S1 to S5 showed an obvious discontinuity in their recorded trajectories (Figure 5a–e), which means that a sudden positional change had occurred during the process of approaching the magnetic source. However, the positional change shrank; for example, the coordinate variation in P1 was (38.28, −19.86) mm for S1, and (26.26, −10.94) mm for S5. Schemes S1 to S4 showed similar trajectories, with an almost constant y coordinate before and after the sudden change moment, while there was a distinct y coordinate decrease in Scheme S5 after the moment. However, for Schemes S6 to S8 (Figure 5f,g), the recorded trajectories of the three magnetic units became smoother, i.e., without positional mutation. The trajectories showed a gradual decrease trend for the entire moving process. P1 variation in the y coordinate shrank: −14.83, −12.64, and −12.54 mm for S6, S7, and S8, respectively.

Regarding the pose information recorded in the dynamic process, the suddenly increased bending angles of Schemes S1 to S5 (Figure 6a–e) were consistent with the corresponding positional change shown in Figure 5. Bending angles *α*_1_ and *α*_2_ of Schemes S1 to S3 always had the same change trend, with the former angle being slightly larger than the latter after the mutation moment. This law started to change in Scheme S4, where bending angle *α*_2_ had a greater increasing trend after the mutation moment and was larger than *α*_1_ (Figure 6d). Then, the new law was more obvious in Schemes S5 to S8 shown in Figure 6f–h. However, the recorded bending angles of the three magnetic units became continuous in Schemes S6 to S8. When considering Schemes S1 to S8, variation in the three bending angles shrank, while the increasing trend of angle *α*_3_ became more apparent.

The above dynamic response of the MMCCRs suggests that the former five schemes adapted to such a scenario needing rapid deformation at a certain moment, while the remainder were useful for a slow and continuous bending application.

### 3.3. Adaptive Navigation of MMCCRs

**Figure 7:**
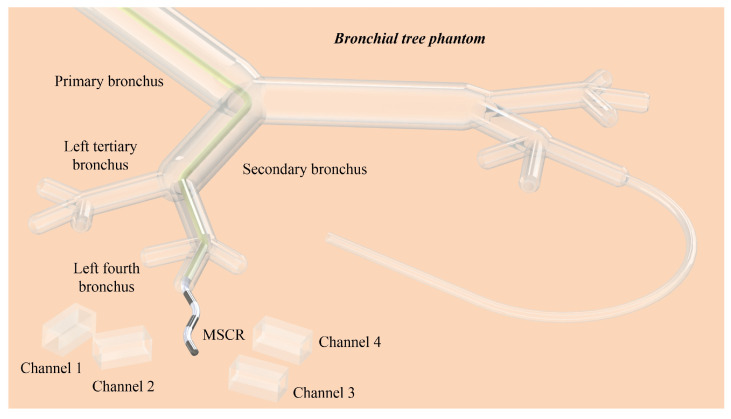
Adaptive multichannel access of the MMCCR.

To demonstrate the adaptive navigation of MMCCRs, a multichannel access experiment was conducted. As shown in Figure 7, an MMCCR with a long soft rear body (yellow tube) passed through the bronchial tree phantom and extended its magnetic head out of the fourth bronchus to access different channels (see dimensional details in Figure S5).

**Figure 8:**
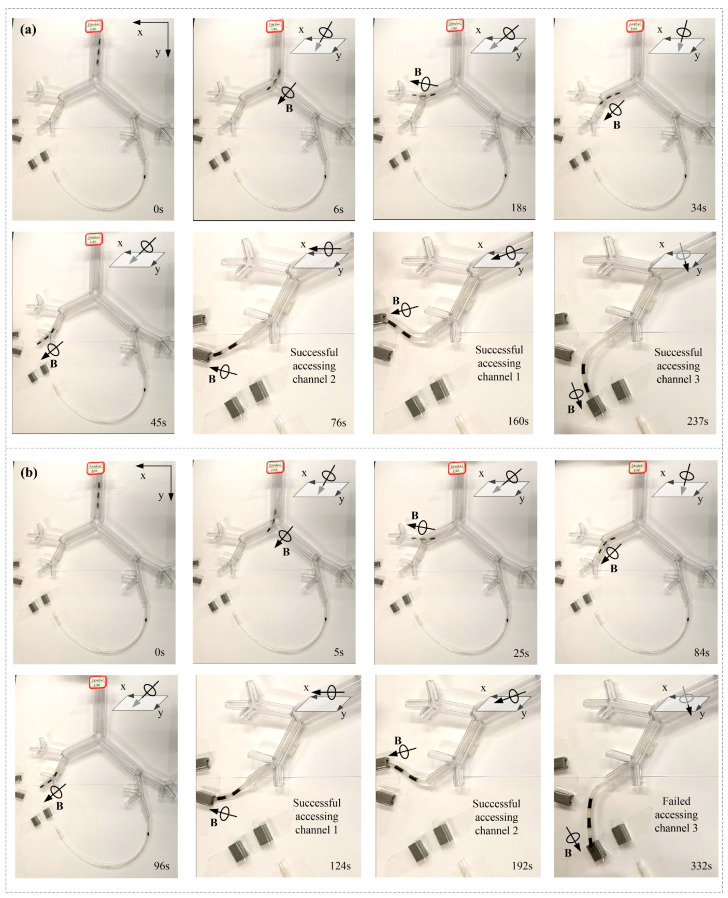
Experimental snapshot of an MMCCR with Schemes (**a**) S8 and (**b**) S1 for accessing different channels.

Schemes S1 and S8 were utilized to conduct this experiment, and an experimental snapshot is shown in Figure 8. A transparent rubber tube with an outer diameter of 3 mm was adhered to the rear end of the MMCCRs with the same nonmagnetic composite. Figure 8a shows that the MMCCR of Scheme S8 was capable of smoothly passing through the bronchial tree phantom and accessing Channels 1–3 (Video S1). Under the applied magnetic actuation, the robot was steered into the left secondary bronchus and the upper left tertiary bronchus at 6 and 18 s, respectively. Then, the robot was retrieved some distance away, and correspondingly steered into the lower left tertiary bronchus and the fourth bronchus at 34 and 45 s, respectively. After several trials, it successfully accessed Channels 2, 1, and 3 in 76, 160, and 237 s, respectively. In Scheme S1, the robot could also be steered inside the bronchial tree phantom and extended out of the fourth bronchus (Figure 8b). However, the S1 robot experienced some difficulty when it was steered into the lower left tertiary bronchus (Video S2) since the applied magnetic field induced a facing-down pose for its tip. Then, the S1 robot was actuated to access Channels 2 and 1, which it achieved in 124 and 192 s, respectively. However, the S1 robot failed to access Channel 3.

**Figure 9:**
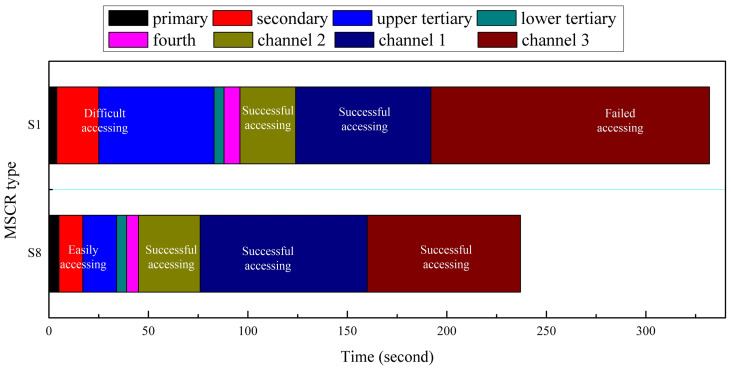
Recorded time slots of two types of MMCCRs for accessing varied channel features.

The recorded time slots of the two types of MMCCRs for accessing various channel features are presented in Figure 9, which shows that the S1 robot spent much more time accessing the upper/lower tertiary bronchus than the S8 did, which corresponded to the facing-down pose induced by magnetic actuation. Regarding accessing Channels 2 and 1, the latter cost a little bit more time than Scheme S1, which may have resulted from the small bending angle compared with that of Scheme S1 under the same actuation level. However, the S1 robot failed to access Channel 3 despite spending more time and having had more trials.

**Figure 10:**
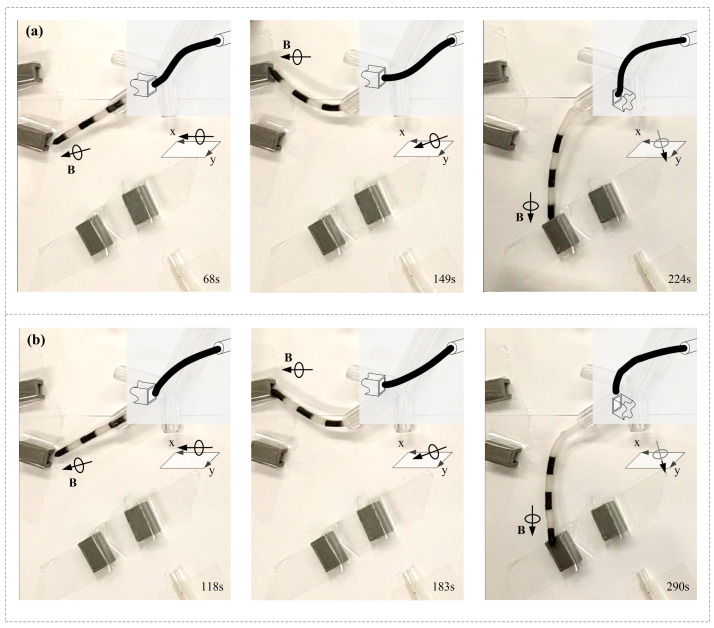
Recorded MMCCR status of Schemes (**a**) S8 and (**b**) S1 before accessing the three channels.

To better understand the channel-access capacity of the two MMCCRs, the robot’s status before the upcoming access is shown in Figure 10. With a magnetic field applied parallel to the *x–o–y* plane, the S8 robot showed a slight S shape when accessing Channels 1 and 2. The slight S shape had also appeared before Channel 3 was accessed under the magnetic field from the *x–o–y* plane. However, when accessing all three channels, the S1 robot presented a single curvature pose under a similar applied magnetic field (Figure 10b). The above deformation of the S1/S8 robot coincided with the calibrated status shown in Figure 4a. The S1 robot was suitable for reaching a larger workspace even though it had a single curvature that could be applied to understand the relatively short period of accessing Channels 2 and 1. The downward S shape of the S8 robot helped it in better adapting to the 10 mm distance between the central bronchial-tree and four-channel planes.

## 4. Discussion

The implementation of biomedical manipulations within the complex and confined channel environments of the body requires the development of small and soft-bodied continuum robots. While most existing soft continuum robots utilize a single response tip for steering, this approach often produces a limited curvature, rendering it be challenging to navigate varied and complex environments. Although the use of multidiscrete response sections could improve the adaptability, reported works have utilized complex fabrication procedures and shown relatively simple deformation styles.

To alleviate the above challenges well, a strategy for quickly obtaining adaptive designs for soft multisectional continuum robots was proposed here. By preprogramming the magnetization directions of the magnetic units and assembling them under certain requirements, an MMCCR could be actuated by the applied magnetic field to achieve a controllable predefined posture and match the task region. The proposed strategy is a simple and general approach for designing task-based soft continuum robots. With this strategy, 8 types of MMCCRs were fabricated and analyzed for the corresponding bending performance. The actuated poses varied, from a single curvature to an S shape, which suggested adaptability to different environments. This adaptability was also demonstrated via the branched channel access experiment. As a proof of concept, this study was first utilized to validate the effectiveness of adaptive MMCCR design and fabrication.

In the future work, more magnetization directions with different angles, ranging from 0 to 90 degrees for the magnetic units, will be considered and designed to obtain more bending statuses. Then, the adaptive assembly of multiple sections with varied magnetic responses (e.g., magnetization-based and material-based) will be considered to achieve smoother access on the basis of the known channel contour. Furthermore, more fabrication methods would be investigated to develop an MMCCR with smaller contours in order to access thinner and more complex channel environments in the body.

A more accurate deformation model for multisectional MMCCRs under magnetic actuation will be established. Then, given a certain assembly of multiple sections with known magnetization directions, deformation performance under an applied magnetic field can be theoretically computed. Conversely, if given a channel environment with known curvature, the combination of magnetic sections can also be theoretically predicted. Furthermore, by designing an MMCCR as a hollow structure and miniaturizing it, it can be integrated with functional surgical microtools to enable versatile manipulations, e.g., microinjection and microablation.

## 5. Conclusions

In this work, we showcased a novel magnetic-polymer-based modular continuum catheter robot (MMCCR) with high adaptability to various environments that could be quickly fabricated using a simple and versatile approach. By preprogramming the magnetization directions of the magnetic units, the assembled multisectional discrete-distributed MMCCR could achieve a range of deformation styles when actuated with a magnetic field. On the basis of the static and dynamic analyses of the MMCCRs, different application scenarios could be predicted, such as steering around sharp turns and navigating inside branches with multiple curvatures. To validate the adaptability of the MMCCRs, we conducted multichannel access experiments using a bronchial-tree phantom. This study significantly contributes to the field of magnetic continuum robots and expands their potential application in the emerging area of biomedical robotics.

## Data Availability

The data supporting the reported results are available via requesting the corresponding author.

## Supplementary Materials

The following supporting information can be downloaded at: https://www.mdpi.com/article/10.3390/mi14050911/s1. Figure S1: schematic of the dimensional design of the MMCR; Figure S2: experimental setup for the characterization of the utilized magnetic field source; Figure S3: image processing procedure for analyzing the bending performance of the robot; Figure S4: mechanical analysis of a typical scheme; Figure S5: dimensional details of the experimental setup for adaptive navigation; Table S1: dimensional parameters of the MMCR; Table S2: magnetization arrangement schemes of the MMCR; Table S3: mechanical parameters of materials used for the MMCR; Video S1: multichannel access experiment via the MMCR with Scheme S8; Video S2: multichannel access experiment via the MMCR with Scheme S1.
