# Supplementary material for: A Fast Soft Continuum Catheter Robot Manufacturing Strategy Based on Heterogeneous Modular Magnetic Units
Source: Micromachines (Basel). 2023 Apr 23;14(5):911. doi: 10.3390/mi14050911 (PMC10223189; doi:10.3390/mi14050911)
Supplement: Supplementary file 1 [file micromachines-14-00911-s001.zip › supplementary material.pdf]

A Fast Soft Continuum Catheter Robot Manufacturing Strategy based on Heterogeneous Modular Magnetic Units

Tieshan Zhang [1, 2, 3, †], Gen Li [1, 2, 3, †], Xiong Yang [3, 4], Hao Ren [1, 2], Dong Guo [1, 2], Hong Wang [3, 4], Ki Chan [5], Zhou Ye [6], Tianshuo Zhao [7], Chengfei Zhang [5], Wanfeng Shang [8,*], and Yajing Shen [3, 4,*]

1 The Robot and Automation Center and the Department of Biomedical Engineering, City University of Hong Kong, Kowloon, Hong Kong 999077, China
2 Shenzhen Research Institute of City University of Hong Kong, Shenzhen 518057, China
3 The Department of Electronic and Computer Engineering, Hong Kong University of Science and Technology, Kowloon, Hong Kong 999077, China
4 Research Center on Smart Manufacturing, Hong Kong University of Science and Technology, Kowloon, Hong Kong 999077, China
5 Prince Philip Dental Hospital, Faculty of Dentistry, University of Hong Kong, Hong Kong 999077, China
6 Applied Oral Sciences and Community Dental Care, Faculty of Dentistry, University of Hong Kong, Hong Kong 999077, China
7 The Department of Electronic and Electronic Engineering, University of Hong Kong, Hong Kong 999077, China
8 Guangdong Provincial Key Laboratory of Robotics and Intelligent System, Shenzhen Institute of Advanced Technology, Chinese Academy of Sciences, Shenzhen 518055, China

* Correspondence: wf.shang@siat.ac.cn (W.S.); eeyajing@ust.hk (Y.S.)

This file includes:

Supplementary text

Supplementary Figures

Supplementary Tables

Supplementary Videos

Supplementary text

The corresponding bending moment of single section (**Figure 3**(a)) can be represented as:

$$M_1 = EI\frac{\alpha_1(l)}{l} \tag{S1}$$

Where $M_1 = \int Fs \cdot dl$. Besides, considering the relationship between the curvature *k* and the deflection angle, i.e., $\kappa = \alpha_1(l)/l = \alpha_1'(l)$, the shear force can be further expressed as:

$$Fs = dM_1/dl\, l = d\left[EI\alpha_1'(l)\right]/dl = EI\alpha_1''(l) \tag{S2}$$

Combining **Equation (S1)** and **Equation (S2)**, the following function about force-induced deflection angle can be obtained.

$$EI\alpha_1''(l) = F_{effect1} l\cos\alpha_1(l) - G_1 l\sin\alpha_1(l) \tag{S3}$$

Taking the boundary conditions $\alpha_1(l=0)=0$ and $\alpha_1'(l=0)=0$ into consideration, **Equation (S3)** can be rewritten as

$$EI\alpha_1(l) = -F_{effect1} l\cos\alpha_1(l) + G_1 l\sin\alpha_1(l) - G_1 l^2 + F_{effect1} l \tag{S4}$$

Supplementary figures

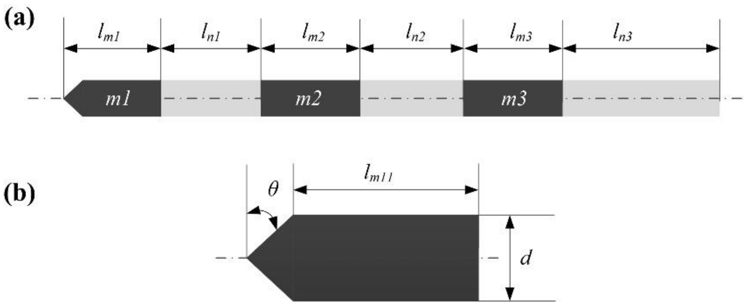

Figure S1. Schematic of the dimensional design of the MMCR

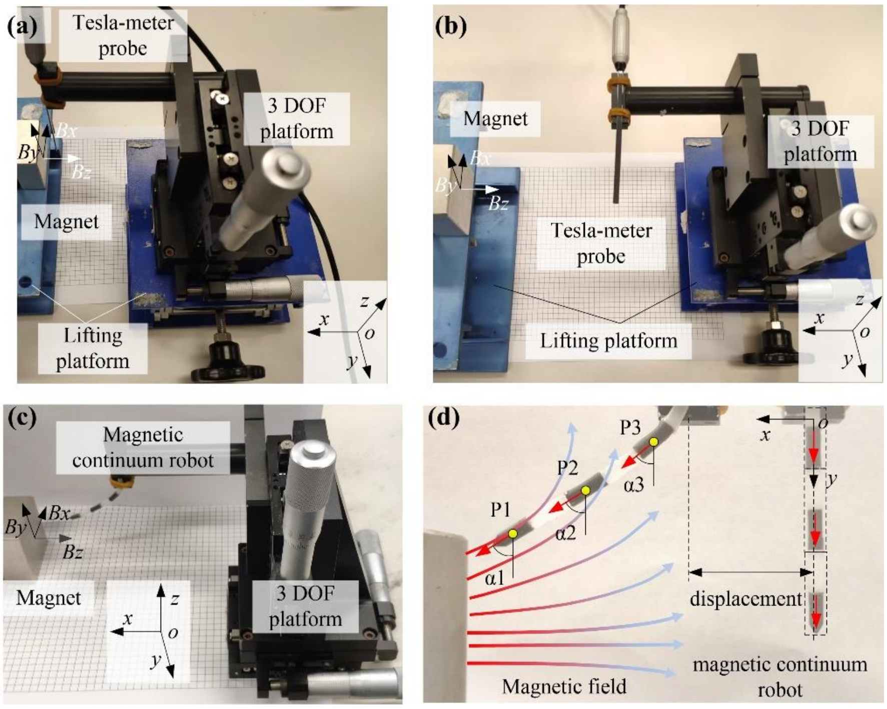

Figure S2. The experimental setup for characterization of the utilized magnetic field source. (a) The setup for measuring the magnetic field distribution along the Bz-axis. (b) The setup for measuring the magnetic field distribution along the By-axis. (c) The experimental setup for evaluating the bending performance of the MMCR under magnetic actuation. (d) The schematic of the position and pose information needed for each magnetic unit.

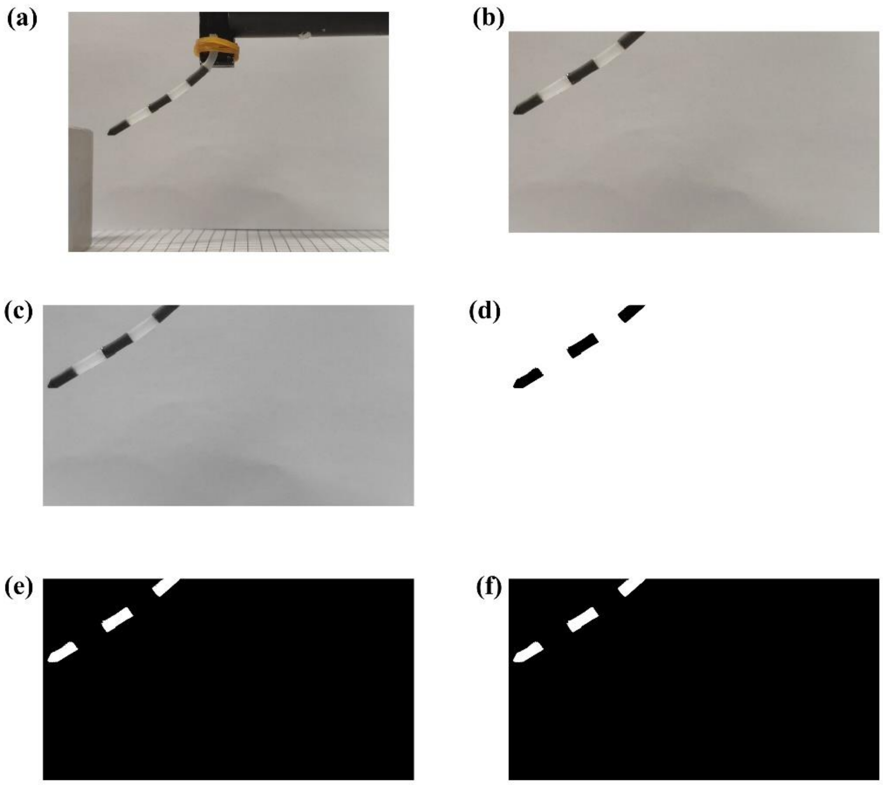

Figure S3. The image processing procedure for analyzing the bending performance of the robot. (a) The recorded bending status of the MMCR. (b) The trimmed image for roughly reducing environmental noise. (c) The grayscale processing for the trimmed image. (d) The binarization processing for the gray image. (e) The inverse processing for the binary image. (f) The edge smoothing processing for the inversed binary image.

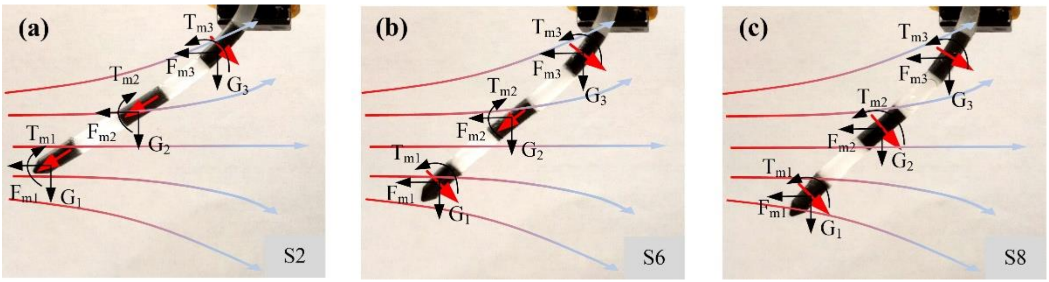

Figure S4. The mechanical analysis of the typical scheme (a) S2, (b) S6, and (c) S8.

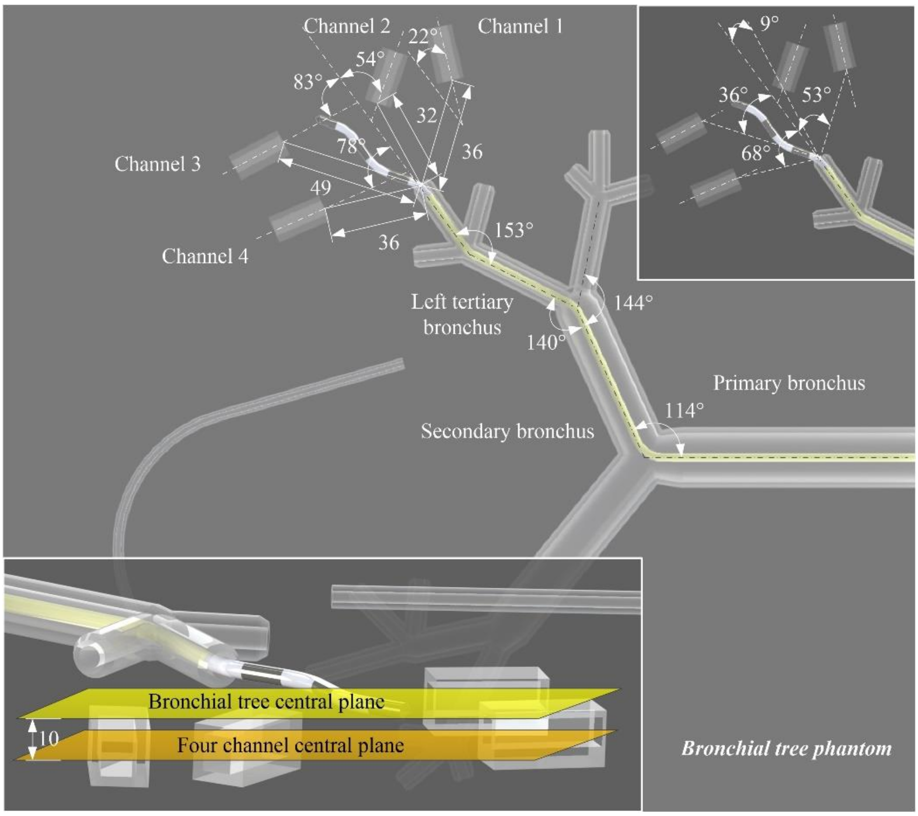

Figure S5. The dimension detail of the experimental setup for adaptive navigation.

Supplementary tables

Table S1. The dimensional parameters of the MMCR

**Symbol**	l_{m1}	l_{m2}	l_{m3}	l_{n1}	l_{n2}	l_{n3}	l_{m11}	d	θ
Value (mm/degree)	8	8	8	8	8	12	6	3	60

Table S2. The magnetization arrangement schemes of the MMCR

**Symbol**	**S1**	**S2**	**S3**	**S4**	**S5**	**S6**	**S7**	**S8**
Magnetization arrangement (m1-m2-m3)	HHH	HHV	HVH	HVV	VHH	VHV	VVH	VVV

Table S3. Mechanical parameters of materials used for the MMCR

**Material**	**Density**	**Elastic module**	**Tensile strength**	**Breaking elongation rate**	**Hardness**
Dragonskin 20	1080 Kg/m^3	337.84 KPa	3.79 MPa	620 %	Shore 20A

Supplementary videos

Video S1. The multi-channel accessing experiment accomplished by the MMCR with scheme S8.
Video S2. The multi-channel accessing experiment accomplished by the MMCR with scheme S1.
